# Molecular Tools for Exploring Polyploid Genomes in Plants

**DOI:** 10.3390/ijms130810316

**Published:** 2012-08-17

**Authors:** Riccardo Aversano, Maria Raffaella Ercolano, Immacolata Caruso, Carlo Fasano, Daniele Rosellini, Domenico Carputo

**Affiliations:** 1 Department of Soil, Plant, Environmental and Animal Production Sciences, University of Naples Federico II, Via Università 100, Portici 80055, Italy; E-Mails: raversan@unina.it (R.A.); ercolano@unina.it (M.R.E.); immacolata.caruso@unina.it (I.C.); carlo.fasano@gmail.com (C.F.); 2 Department of Applied Biology, University of Perugia, Borgo XX Giugno 74, Perugia 06121, Italy; E-Mail: roselli@unipg.it

**Keywords:** genome doubling, molecular markers, microarrays, gene expression, next generation sequencing, transcriptomics

## Abstract

Polyploidy is a very common phenomenon in the plant kingdom, where even diploid species are often described as paleopolyploids. The polyploid condition may bring about several advantages compared to the diploid state. Polyploids often show phenotypes that are not present in their diploid progenitors or exceed the range of the contributing species. Some of these traits may play a role in heterosis or could favor adaptation to new ecological niches. Advances in genomics and sequencing technology may create unprecedented opportunities for discovering and monitoring the molecular effects of polyploidization. Through this review, we provide an overview of technologies and strategies that may allow an in-depth analysis of polyploid genomes. After introducing some basic aspects on the origin and genetics of polyploids, we highlight the main tools available for genome and gene expression analysis and summarize major findings. In the last part of this review, the implications of next generation sequencing are briefly discussed. The accumulation of knowledge on polyploid formation, maintenance, and divergence at whole-genome and subgenome levels will not only help plant biologists to understand how plants have evolved and diversified, but also assist plant breeders in designing new strategies for crop improvement.

## 1. Polyploidy in the Plant Kingdom: Occurrence and Significance

The genome sizes of eukaryotes can differ 10,000-fold and part of these differences may be attributed to changes in the ploidy level. Polyploids are organisms having more than two complete sets of chromosomes in their cells. They are common in angiosperms, where at least 70% of the species experienced one or more events of genome doubling during their evolutionary history [1,2]. Many crop species are polyploids (Table 1), and it was stated that “life on earth is predominantly a polyploid phenomenon and civilization depends mainly on use of polyploid tissues—noteworthy is the endosperm of cereals” [3]. Polyploidization is considered a major evolutionary force in plants. It is a definitive cause of sympatric speciation due to the immediate reproductive isolation between newly formed polyploids and their parents [4]. Polyploidization also makes it possible to overcome hybrid sterility and produce viable offspring following interspecific hybridization. However, there are still several open questions related to polyploidy and polyploidization. For example, Soltis *et al*. [5] reported that polyploidy frequency in angiosperms is high, even if the number of lineages that really derived from genome-wide duplication (WGD) events is still largely unknown. Similarly, it is not clear if polyploidy causes a change in the interaction with herbivores and pollinators.

Based on their origin, polyploids are classified into two major groups, autopolyploids and allopolyploids [6]. The former are the result of doubling homologous genomes (e.g., autotetraploid AAAA) from a single, or closely related, species. The latter are the result of hybridization between different species. Therefore, they combine two or more different genomes (e.g., allotetraploid AABB). As a consequence, autopolyploids may form multivalents at meiosis, and have polysomic inheritance. By contrast allopolyploids show bivalent pairing and have disomic inheritance [7]. There are several mechanisms that may lead to an increase in ploidy level in plants. However, there is strong circumstantial evidence that sexual polyploidization through gametes with unreduced chromosome number (2*n* gametes) represents the main route for polyploidization [8,9]. 2*n* gametes generally result from the expression of mutations affecting micro- and megasporogenesis. Such mutations have been extensively studied in a number of genera, including *Solanum*, *Medicago*, *Manihot*, *Malus*, *Arachis*, *Lolium*, and *Agropyrum*, and have been generally attributed to the action of single recessive genes [10]. The first gene (*AtPS1*) involved in 2*n* gamete production has been identified in *A. thaliana* [11]. *AtPS1* mutants display an anomalous (parallel, fused, tripolar) orientation of spindles at metaphase II of male meiosis, leading to the production of 2*n* pollen. Similarly, 2*n* gametes have been observed in the *jason* (*jas*) [12], *switch1(swi1)/dyad* [13,14], *osd1* and *tam* (*CYCAS1;2*) mutants [15,16].

**Figure 1:**
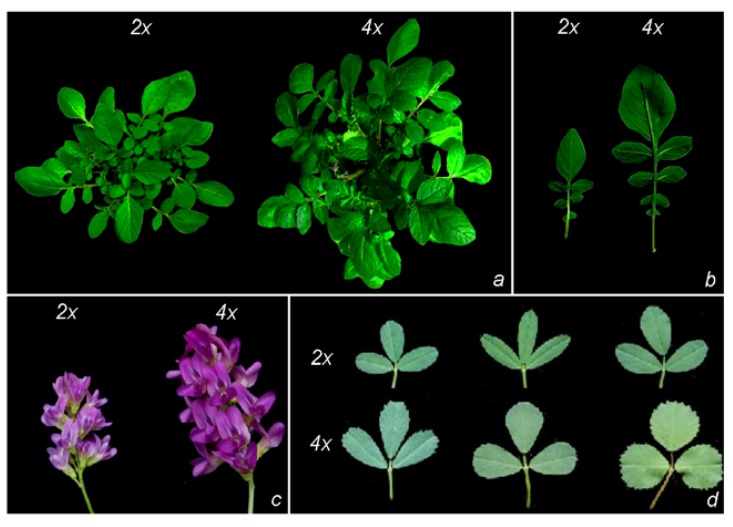
Phenotypic variation between diploids and tetraploids in *Solanum commersonii* (**a**, **b**) and in *Medicago sativa* (**c**, **d**). A diploid (2*n* = 2× = 24) clone of *S. commersonii* was subjected to oryzaline treatment, an antimitotic drug commonly employed to induce chromosome doubling in plants. The autotetraploid (2*n* = 4× = 48) genotype displayed larger size at both whole plant (**a**) and leaf (**b**) level. (**c**) Diploid *M. sativa* subsp. *coerulea* (2*n* = 2× = 16, left) and its cultivated tetraploid counterpart, *M. sativa* subsp. *sativa* (2*n* = 4× = 32, right) differ clearly for flower size. (**d**) Leaves of diploid (upper row) and tetraploid (bottom row) plants obtained from crossing two diploid *M. sativa* plants producing both *n* and 2*n* gametes. Leaves are the best qualitative component of forage: tetraploid *M. sativa* has larger leaves and is cultivated.

In a given species and habitat, the acquisition of the polyploid condition may bring about several advantages. Following polyploidization, often novel phenotypes appear or variation exceeds the range observed in the diploid parental species. Fawcett *et al.* [17] suggested that polyploids had a better chance to survive the Cretaceous-Tertiary extinction event. Phenotypic advantages may include, among the others, changes in morphology, physiology and secondary metabolism that confer an increased fitness. Some of these traits, such as increased drought tolerance, pathogen resistance, longer flowering time, larger vegetative and reproductive organs (Figure 1) may represent important plant breeding targets and, therefore, increase the potential use of polyploids in agriculture. From a genetic point of view, the most significant advantages associated with polyploidy are probably heterosis and gene redundancy [18]. Heterosis is due to non-additive inheritance of traits in a newly formed polyploid compared to its parents. Notably, it can be present also at the gametophytic level. The main factors that affect non-additive inheritance are likely novel regulatory interactions and allelic dosage [19]. Gene redundancy promotes neofunctionalization of duplicated genes, in the long term, but also immediately protects against deleterious recessive alleles. In a recent treatment, Mayrose *et al*. [20] shed more light on the evolutionary dynamics and consequences of polyploidy. The authors, computing the net diversification rates of polyploid lineages for ferns, lycophytes, gymnosperms and angiosperms, hypothesized that polyploidy is often an evolutionary dead end. However, the possible longer-term evolutionary success of those polyploids that survive, needs to be tested. Several studies provided evidence that extensive and reproducible genetic and epigenetic changes are possible following polyploidization [9]. They include DNA and histone methylation, DNA elimination, gene losses, gene neo- and subfunctionalization, translocations, amplification and reduction of repetitive sequences and alteration of gene expressions through different mechanisms (methylation-mediated silencing, transposon activation, intergenomic interactions, *etc*.). Given the recent advances in the field of plant molecular biology and biotechnology, through this review we provide an overview of the most suitable technologies and strategies that can allow, at the molecular level, efficient studies on polyploid plants, possibly promoting research in areas that have been ignored or underestimated so far. Reference to some recent significant findings will also be made.

## 2. Methods for Genome Analysis

A combination of genetic mapping, molecular cytogenetics, sequence and comparative analysis has shed new light and opened perspectives on the nature of ploidy evolution at all timescales, from the base of the plant kingdom, to intra- and interspecific hybridization events associated with plant domestication and breeding. Strong evidences on the mechanisms of genomic modification have come from the use of physical analysis of chromosomes by *in situ* hybridization techniques and from genome-wide molecular marker analyses.

### 2.1. *In Situ* Hybridization

*In situ* hybridization represents the bridge between the chromosomal and molecular level of genome investigation. In recent years it has received a renewed interest for detecting chromosome rearrangements. It is very powerful for reliable identification of chromosomes, allowing the positioning of unique sequences and repetitive DNAs along the chromosome(s). Fluorescent *in situ* hybridization (FISH) is based on fluorescent labels linked to DNA probes and visualized under a fluorescence microscope. Genomic *in situ* hybridization (GISH) involves the use of total genomic DNA of species as a probe on chromosomes, thus leading to whole genome discrimination rather than the localization of specific sequences. There are several examples on the use of these techniques. Studies on the distribution of four tandem repeats in allotetraploids *Tragopogon mirus*, *Tragopogon miscellus* and their diploid parents provided evidence that chromosomal rearrangements did not occur following polyploidization, as suggested by the additive patterns of polyploids [21]. By contrast, in newly synthesized allotetraploid genotypes of *B. napus*, Szadkowski *et al.* [22] demonstrated extensive genome remodeling due to homeologous pairing between the chromosomes of the A and C genomes. Based on high-resolution cytogenetic maps, Wang *et al.* [23] demonstrated that genome size difference between the A and D sub-genomes in allotetraploid cotton was mainly associated with uneven expansion or contraction between different regions of homoeologous chromosomes. Recently, Chester and co-workers [24] combined GISH and FISH analysis to demonstrate that in natural populations of *T. miscellus* extensive chromosomal variation (mainly due to chromosome substitutions and homeologous rearrangements) was present up to the 40th generation following polyploidization.

### 2.2. Molecular Marker-Based Genetic Mapping

Genetic mapping in polyploids presents unique problems with respect to diploid species, since segregations and statistical methods are much more complicated, and large segregating populations are needed to obtain reliable genetic distance estimates [25]. Important results were obtained using classical Restriction Fragment Length Polymorphism markers (RFLP) in *B. napus* [22,26–28], *Draba norvegica* and *T. miscellus* [29], and wheat [30] or employing Amplified Fragment Length Polymorphism (AFLP) in *Arabidopsis* [31], *Brassica* [32], and *Spartina* [33].

Autopolyploids are particularly intractable because segregation depends on chromosome pairing behavior (preferential *vs*. random pairing) and double reduction [34]. A simplification that has been generally adopted for mapping dominant molecular markers is to only utilize the so-called single dose markers from each parent, *i.e.*, those segregating 1:1 in the mapping population (for example, a population obtained from the cross Mmmm × mmmm in a tetraploid species) [35,36]. Statistical models for QTL mapping have been developed for polyploids with bivalent pairing, taking into account preferential *vs*. random chromosome pairing behavior by incorporating a preferential pairing factor [37,38]. Molecular marker segregation allows estimating chromosome pairing behavior. For instance, significant preferential pairing was evidenced in a population obtained by crossing *M. sativa* subsp. *falcata* × *M. sativa* subsp. *sativa* autotetraploid genotypes [39], whereas the data of Julier *et al.* [40] did not support preferential pairing in a mapping study involving cultivated *M. sativa* genotypes. In *Salix*, an allotetraploid origin was inferred based on molecular marker segregation supporting preferential pairing [41]. The recourse to diploid species in search of synteny can facilitate genetic mapping of agronomic traits in complex polyploids [42].

Recently, improvements have been proposed in polysomic inheritance analysis through the determination of the number of allele copies in microsatellite loci [43,44]. In particular, a method named Microsatellite Allele Dose and Configuration Establishment (MADCE) was proposed to establish the exact dose and configuration of microsatellite alleles in allo-octoploid cultivated strawberry (*Fragaria × ananassa*) [44]. This technique enhances the utility of SSR markers in polyploids by overcoming the limitation of single dose markers. A recently introduced type of molecular marker, Diversity Arrays Technology (DArT), was applied to mapping in a tetraploid recombinant inbred line population obtained by crossing wild *Avena magna* (2*n* = 4× = 28) with cultivated *A. sativa* (2*n* = 6× = 42) [45].

### 2.3. Methylation Sensitive Molecular Markers

The use of an AFLP-like method using restriction enzymes sharing the same recognition site but having differential sensitivity to DNA methylation (isoschizomeres) was proved to be efficient and reliable for the determination of genome-wide DNA methylation patterns [46]. This technique, termed Methylation-Sensitive Amplified Polymorphism (MSAP), is based on the use of the isoschizomers *Hpa*II and *Msp*I both recognizing the 5′CCGG sequence, but affected by the methylation state of the outer or inner cytosine residues. Using this method, noteworthy results were obtained in newly synthesized polyploids. In *Arabidopsis*, Madlung *et al.* [47] demonstrated that frequent changes occurred in F_4_ allotetraploids when compared with the parents. Changes involved both increases and decreases in methylation, but no overall hyper- or hypomethylation. Similarly, alterations in cytosine methylation in wheat occurred in about 13% of the loci, either in F_1_ hybrids or in allopolyploids. Notably, alterations in methylation patterns affected both repetitive DNA sequences and low-copy DNA in approximately equal proportions [25]. On the other hand, lack of rapid DNA methylation changes at symmetric CCGG sites was hypothesized in allopolyploid cotton [48]. A similar behavior was observed also in *Brassica* [49] and sugarcane [50].

### 2.4. Comparative Genome Analysis

Comparative genomics research has gained importance as a powerful tool for addressing both fundamental and applied questions in genome evolution [51–53]. The implementation of these methodologies, however, requires consideration of the variable rates at which different aspects of genome evolution occur [52]. Comparing different wheat species, genomic rearrangements originating by illegitimate DNA recombination were identified as a major evolutionary mechanism [54,55]. Innes *et al.* [56], comparing homologous regions in several related legume species, demonstrated that retroelements were the largest contributor to duplicated regions. Comparative analysis of *Brassica oleracea* triplicated segments showed that 35% of the genes were lost. Retained genes were dosage-sensitive and not randomly located. Duplicates of transcription factors and members of signal transduction pathways were significantly over-retained following WGD, whereas these same functional gene categories exhibited lower retention rates following smaller scale duplications [57]. For instance, in four independent polyploid wheat lineages, recurrent deletions of Puroindoline (*Pin*) gene at the grain *Hardness* (Ha) locus were identified [58].

Phylogenetic and taxonomic studies have been conducted in order to pinpoint the exact placement of the ancient polyploidy events within lineages and to determine when novel genes resulting from polyploidy have enabled adaptive processes. Recent genomic investigations not only indicated that polyploidy is ubiquitous among angiosperms, but also suggested several ancient WGD events [59–62], even in basal angiosperm lineages. Phylogenetic reconstruction with completely sequenced genomes suggested that genome doubling led to a dramatic increase in species richness in several angiosperms, including *Poaceae*, *Solanaceae* and *Brassicaceae*, thus contributing to the dominance of seed plants and angiosperms [63]. To date, only a few reports investigated the fate of the genome after polyploid formation [64]. The probability of fixation and maintenance of duplicated genes depends on many variables. Transposable elements may play a key role in fuelling genome reorganization and functional changes following allopolyploidization. A pivotal example of using comparative approaches to investigate the role of retroelements in polyploids is provided by Wawrzynski *et al*. [65]. The authors, investigating the nonautonomous retrotransposon replication in soybean estimated a much greater impact of such transposable elements on genome size than previously appreciated. More recently, in wheat Kraitshtein *et al*. [66] reported a retrotransposition bursts in subsequent generations. By contrast, no evidence for a transposition burst was found in different allopolyploid species [31,67,68]. Comparative approaches in which genetic events are considered both in a phylogenetic and genetics framework should be conceptualized and modeled.

### 2.5. High-Throughput DNA Sequencing and High Resolution Melting (HRM) Analysis

High-throughput DNA sequencing associated with computational analysis provides general solutions for the genetic analysis of polyploids [69]. However, ploidy is a substantial challenge in sequencing and assembly of plant genomes. A number of biological factors influence the feasibility of discrimination, including the degree of gene family complexity, and the reproductive system. Of course, the level of knowledge concerning the progenitor diploid species is also very important. To date, all attempts to sequence polyploids have relied on either a reduction in ploidy or a physical separation of chromosomes. Attempts to sequence a heterozygous diploid potato genome (RH89-039-16) were challenging due to the high degree of heterozygosity [70]. In order to bypass the difficulties of sequencing the polyploid genome of cultivated strawberry (*Fragaria x ananassa*), woodland strawberry (*Fragaria vesca*) was sequenced [71]. In *B. napus*, the polyploidy issue was addressed by sequencing leaf transcriptome across a mapping population and representative ancestors of the parents of the population [72]. The Wheat Genome Initiative (http://www.wheatgenome.org/) has focused on flow cytometry separation of individual or groups of homeologous chromosomes [73]. To better understand the nature and extent of variation in functionally relevant regions of a polyploid genome, a sequence capture assay to compare exonic sequences of allotetraploid wheat accessions was developed [74]. In cultivated wheat gene duplications were predominant, while in wild wheat mainly deletions were identified. Exon capture proved to be a powerful approach for variant discovery in polyploids. This technique has the potential to identify variation that can play a critical role in the origin of new adaptations and important agronomic traits.

A wealth of SNP detection approaches has been applied to study polyploidy in plants. Akhunov *et al.* [75], using the Illumina GoldenGate assay, identified a high number of SNPs in tetraploid and hexaploid wheat. More recently, Allen *et al*. [76] from Illumina GAIIx data identified more than 14,000 putative SNPs in 6225 distinct hexaploid bread wheat reference sequences. In elite inbred maize lines, more than 1 million SNPs have been identified an Illumina sequencing platform [77]. In the heterozygous polyploid sugarcane, a targeted SNP discovery approach based on 454 sequencing technology was developed by Bundock *et al.* [78]. Using a 454 and Illumina expressed sequence tag sequencing of the parental diploid species of the allotetraploid *Tragopogon miscellus*, Buggs *et al*. [79] identified more than 7,700 SNPs differing between the two progenitor genomes the allotetraploid derived from. The Sequenom MassARRAY iPlex platform [80] was used by to validate 92 SNP markers at the genomic level that were diagnostic for the two parental genomes. SNP discovery was also pursed through 454 technology coupled to High Resolution Melting (HRM) curve analysis in tetraploid alfalfa (*Medicago sativa*) [81]. HRM is a technique that can identify mismatches, even for single bases, in amplicons containing heteroduplex molecules [82], and is emerging as a powerful tool for polyploid genetics [83]. It was demonstrated that the 454 system is a cost-effective approach for SNP discovery targeted to genes of interest in polyploid genomes, and that HRM can identify different alleles in polyploids [66,68]. Salmon *et al.* [84] detected homoeologous SNPs in *G. arboreum* (A genome), *G. raimondii* (D genome), and *G. hirsutum* (AD genomes). The authors estimated that the proportion of genome in *G. hirsutum* that has experienced non reciprocal homoeologous exchanges since the origin of polyploid cotton 1–2 Mya was between 1.8% and 1.9%. SNPs have also been discovered in transcriptome sequences of polyploidy *B. napus* [85]. Next-generation sequencing has been used to mine SNPs in elite wheat germplasm [76].

## 3. Methods for Gene Expression and Regulation Analysis

Several methods have been developed for quantifying gene transcription and regulation patterns in polyploids. Although studying gene expression changes in allopolyploids is more complicated than in autopolyploids, most studies on ploidy-related gene expression changes were carried out on synthetic allopolyploids. Indeed, genome merger and doubling can determine widespread transcriptome modifications, generating cascades of novel expression patterns, regulatory interactions, and new phenotypic variations that subsequent natural selection may act upon.

### 3.1. Northern Hybridization and cDNA-AFLP

When sequence information was still scanty, comprehensive transcript-profiling for quantitatively measuring gene expression variation was carried out by Northern blot analysis. This technique involves the use of electrophoresis to separate RNA samples and detection with a labeled probe complementary to a specific RNA target sequence. There are only a few examples on the use of this type of analysis in polyploids. It was used by Guo and co-workers [86] to investigate the dosage effects of 18 genes in an autopolyploid maize series (1×, 2×, 3×, and 4×). Expression levels of genes were dependent on chromosomes dosage, although some varied their expression in response to the “odd” or “even” ploidy. By contrast, several examples are available on the use of cDNA-AFLP. It is a PCR-based technique, which relies on digestion of cDNA by two restriction enzymes and ligation of specific adapters. A set of specific primers designed for these adapters allow simultaneous amplification of fragments under stringent conditions. In synthetic allotetraploids between A. thaliana and A. arenos*a*, Comai *et al.* [87] found 20 suppressed genes out of 700 examined. Similarly, Lee and Chen [88], by extending the analysis also to *A. suecica* (a natural allopolyploid likely formed through pollination of *A. arenosa* with 2*n* gametes from *A. thaliana*) were able to identify a set of 10 different genes differentially expressed in *A. suecica* and its progenitors. In synthetic *Triticum aestivum* allohexaploids [89], about 8% of transcripts displayed altered expression, and >95% of them were reduced or absent. Similar gene expression changes have been found in cotton allopolyploids [90]. Another example on the use of this technique is offered by the work carried out by Tate *et al.* [91]. In newly synthesized *Tragopogon* allotetraploids, preferential expression of parental homeologues displayed a correlation with a loss of parental genomic fragments. Notably, such changes were not observed in newly developed *Tragopogon* F_1_ hybrids, implying that they arose following genome duplication.

### 3.2. Single-Strand Conformational Polymorphism (SSCP) Analysis

This technique detects sequence variations (single-point mutations and other small-scale DNA changes) through electrophoretic mobility differences. DNA that contains a sequence mutation (even a single base pair change) displays a different measurable mobility compared to reference DNA when electrophoresed in non-denaturing, or partially denaturing conditions. Due to these features, SSCP was employed to distinguish between homoeologous cDNA molecules. This approach has been applied to *A. suecica* [88], cotton [90] and wheat [92] leading to the finding that, basically, genes duplicated by polyploidy are rarely expressed at similar levels, and that there is a biased expression or silencing of some homeologous gene pairs.

### 3.3. Microarrays

Technologies to monitor gene expression achieved a breakthrough through the introduction of microarrays [93]. Genome and transcriptome sequencing have speed up probe development, which consequently resulted in the commercial availability of whole-genome microarrays for many model and crop species. This also gave the possibility to design custom arrays at affordable costs. Approaches of comparative expression profiling have mainly focused on synthetic allotetraploids revealing both additive and non-additive gene expression. The former occurs when gene expression level in the tetraploid is either the sum of the parental values or equal to the mid-parent value (MPV). For instance, tanscriptional profiling of resynthesized *A. suecica* lines from newly created autotetraploid *A. thaliana* and the natural tetraploid *A. arenosa* revealed that, albeit most of genes were additively expressed (from 65% to 95%), more than 1,400 genes diverged from the MPV. The combination of diverged parental genomes in a common nucleus during allopolyploidization implies the reunion of previously diverged regulatory hierarchies, which likely entails non-additive gene expression. This hypothesis has been validated by genome-wide expression analyses also in synthetic polyploids of wheat, cotton, *Senecio*, *Brassica*, and *Spartina* [94–99]. These studies demonstrated that allopolyploid plants exhibit considerable transcriptome alterations as compared with their diploid progenitors. Transcriptome analyses of autopolyploids suggested that there are less dramatic alterations of gene expression compared to allopolyploids [100]. Expression profiling analyses of autotetraploid *A. thaliana* of two different accessions revealed that transcriptome alterations caused by autopolyploidy depend on genome or genetic composition [101]. Microarray analysis provided evidence that ~10% of the ~9,000 potato genes tested displayed expression changes (within the twofold level) among a potato autopolyploid series (1×, 2×, and 4×) [102]. A similar twofold level change was detected in a corn ploidy series (1×–4×) [103].

DNA microarray technology has been also used to profile expression of noncoding RNA molecules naturally occurring in the plant genomes, such as micro RNA (miRNA). They are a class of 20–24 nucleotide small RNAs that repress their target genes by mRNA degradation or translational repression. Therefore, identification and quantification of miRNAs is deemed essential to understanding an organism’s or tissue’s gene regulatory network [104]. MicroRNA expression profiling was performed with custom designed chips in both natural *A. suecica* and resynthesized *Arabidopsis* genotypes. It indicated that many miRNA and trans-acting siRNA (tasiRNA), *i.e.*, endogenous siRNAs that direct the cleavage of non-identical transcripts, are non-additively expressed [105]. Among the differentially expressed miRNAs, miR163 is severely repressed in leaves and flowers of *A. arenosa* and allotetraploids, but is highly expressed in *A. thaliana*. Analysis by Ng *et al.* [106] demonstrated that miR163 expression differences results from cis-acting effects, as well as from trans-acting repressor(s) that are present in *A. arenosa* and allotetraploids but absent in *A. thaliana.*

### 3.4. High Throughput RNA Sequencing

Next-generation sequencing (NGS) technologies are changing the ways in which gene expression is studied. The principle behind these applications of high throughput sequencing technologies, which have been termed RNA-seq, is simple: complex RNA samples are directly sequenced to determine their content. Therefore, unlike hybridization-based data requiring the estimation of RNA amount by image analysis, RNA-seq data consists of absolute numbers of reads from each gene. These data are highly suitable for the analysis of gene expression since, not relying on probes, they are less error-prone than previous methods and allow to determine absolute expression levels [107]. Sequencing-based methods also permit the genome-wide study of small RNA expression. In *T. miscellus* allopolyploids, Buggs *et al*. [79] profiled almost 3000 SNP markers using an Illumina RNA-seq approach to study differential expression of duplicate homologous genes derived from the parental genomes. The authors found expression biases among tissues in the diploid parents (*T. dubius* and *T. pratensis*) in comparison to the natural allopolyploids, as well as uniform expression in F_1_ and first-generation synthetic allopolyploids. To explain the observed “transcriptomic shock”, they hypothesized a loosening of gene expression regulation, which may set the stage for gradual evolution of novel patterns of expression in the early generations of polyploidy. Croate and Doyle [108] used quantitative reverse transcriptase-polymerase chain reaction and RNAseq in allopolyploid *Glycine dolichocarpa* and its diploid progenitors. They inferred dosage responses for several thousand genes and showed that most of them had partial dosage compensation. In *G. max*, RNA-seq allowed the identification of the gene family likely contributing to differences in photosynthetic rate between the allotetraploid and its progenitors [109]. The authors also provided evidences that the tetraploid appeared to use the “redundant” gene copies in novel ways. In *A. thaliana* allopolyploids, transcriptome profiling was carried out by Ha and colleagues [105] through high-throughput cDNA pyrosequencing. For the first time, these authors gained insight into small RNA expression diversity and evolution in closely related species as well as in interspecific hybrids. The data suggested a role for small RNAs in buffering against genomic shock in *Arabidopsis* interspecific hybrids and allopolyploids. In particular, they seem to have a central role in maintaining genome and chromatin stability as well as in modulating non additive gene expression. In addition, Ha *et al*. [105] found that repeat- and transposon-associated siRNAs (rasiRNA and TE-siRNA, respectively) were highly divergent between *A. thaliana* and *A. arenosa* and their non additive gene expression in allopolyploids were not correlated. By contrast, miRNA and tasiRNA sequences were conserved between species, but their expression patterns were highly variable between the allotetraploids and their progenitors.

## 4. Methods for Protein Analysis

Compared with genomic and gene expression variations, changes in proteins and gene products in polyploids and their progenitors were rarely examined. An early study on genome-wide protein profiling was performed in maize lines of different ploidies by sodium dodecyl sulfate–polyacrylamide gel electrophoresis (SDS–PAGE) [110]. This is a technique for the separation of proteins according to their molecular weight, in the presence of a reducing agent (2-mercaptoethanol). Data obtained showed that expression per genome for most maize proteins did not change with ploidy, even though ploidy-modulated expression changes were detected for a few proteins. A more powerful method to analyze complex protein mixtures is the protein two-dimensional electrophoresis (2-DE) analysis, in which proteins are separated according to their isoelectric point and mass. In diploid, tetraploid and hexaploid wheat 2-DE experiments showed that the expression of homeologous proteins in hexaploid wheat depended on interactions among the parental A, B and D genomes [111]. Other recent studies using 2-DE indicated numerous and unbiased variations of proteins in newly synthesized *B. napus* [112,113] and wheat hybrids [114]. Using protein 2-DE coupled with mass spectrometry (MS) assays, a recent study in maize showed a positive correlation of differentially expressed proteins with ploidy levels [115]. The highest correlations were found in diploid–hexaploid and tetraploid–hexaploid comparisons. Recently, Ng and co-workers [116] were able to study quantitative changes in the proteome of *Arabidopsis* autopolyploids and allotetraploids and their progenitors using the isobaric tags for relative and absolute quantification (iTRAQ) technique, coupled with mass spectrometry. The levels of protein divergence ranged from ~18% between *A. thaliana* and *A. arenosa* to ~7% between an *A. thaliana* diploid and autotetraploid. In F_1_- and F_8_-resynthesized allotetraploids the proteomic divergence relative to MPV was intermediate (~8%). These data suggest that, during polyploidization, rapid changes occurring in post-transcriptional regulation and translational modifications of proteins can lead to high protein discrepancy between species.

## 5. Conclusions and Perspectives

With the speed of technology improvement and the application of genomic tools, polyploidy research is undergoing a renaissance. It can be expected that comprehensive studies using multidisciplinary approaches will push the boundaries of current methodologies to translate the knowledge gained into practical applications. Particularly significant will be the high-throughput genome-wide approaches to unraveling the genetic and epigenetic consequences of polyploidization and the availability of phenotyping platforms. They are all reaching an unprecedented level of resolution at relatively affordable costs to the point that genotyping-by-sequencing [117] and targeted sequence capture [118] are now feasible also for high diversity, large genome species. NGS not only will extend the possibilities of gene and marker discovery, but will enable genome-wide quantification of gene expression. It will also allow direct genome-scale investigation of chromatin and DNA methylation cross-talk, by ChIP-Seq, bisulfite sequencing, *etc*. Characterizing transcripts through sequencing is advantageous to circumvent problems posed by highly redundant and extremely large genomes. It should be pointed out that the rapid pace at which new sequencing technologies are emerging is generating a growing disparity between the rate of data generation and its full and biologically meaningful analysis. However, there are outstanding examples addressing successful strategies for dealing with these challenges [109,118–120].

Genetic mapping can exploit robust statistical models, and will be crucial for identifying the genes underlying the polyploidization process in the bulk of the fast growing genome sequence information. Merging results from genetic, genomics and proteomics investigations will help to understand to what extent polyploid genome flexibility is associated with amplified responses to selection. We have recently hypothesized that defense response plasticity of potato could be correlated to gene number and category and cluster organization [121]. Understanding polyploid evolution requires knowledge to be integrated at the population level, and will have not only to rely on suitable experimental designs, but also on surveys of variation at multiple levels. Recent and forthcoming sequencing technologies are providing a wealth of genomic data to be released soon, also for wild species that can be employed for evolutionary studies. Until recently, sequencing complex genomes was considered very challenging due, for example, to the difficulties in discriminating among paralogous, hortologous, and homoeologous sequences. However, the availability of the genome sequence of the ancestors, the reduction in the ploidy level or the physical separation of chromosomes offered the possibility to circumvent these challenges and examples of polyploid genomes fully sequenced have become available [122,123]. The accumulation of knowledge on polyploid formation, maintenance, and divergence at the whole-genome and subgenome levels will not only help plant biologists to understand how plants have evolved and diversified, but also assist plant breeders in designing new strategies for crop improvement.

## Figures and Tables

**Table 1:** Examples of polyploid crops. The somatic chromosome number is reported in brackets.

Crop	Species
Cereals	*Triticum aestivum* (6× = 42); *T. durum* (4× = 28); *Avena sativa* (6× = 42); *A. nuda* (6× = 42)
Forage grasses	*Dactylis glomerata* (4× = 28); *Festuca arundinacea* (4× = 28); *Agropyrum repens* (4× = 28); *Paspalum dilatatum* (4× = 40)
Legumes	*Medicago sativa* (4× = 32); *Lupinus alba* (4× = 40); *Trifolium repens* (4× = 32); *Arachis hypogaea* (4× = 40); *Lotus corniculatus* (4× = 32); *Glycine max* (4× = 40)
Industrial plants	*Nicotiana tabacum* (4× = 48); *Coffea* spp. (4× = 44 fino a 8×); *Brassica napus* (4× = 38); *Saccharum officinalis* (8× = 80); *Gossypium hirsutum* (4× = 52)
Tuber plants	*Solanum tuberosum* (4× = 48); *Ipomoea batatas* (6× = 96); *Dioscorea sativa* (6× = 60)
Fruit trees	*Prunus domestica* (6× = 48); *Musa* spp. (3× = 33; 4× = 44); *Citrus aurantifolia* (3× = 27); *Actinidia deliciosa* (4× = 116); *P. cerasus* (4× = 32)

## References

[1] Masterson J. (1994). Stomatal size in fossil plants: Evidence for polyploidy in majority of angiosperms. Science.

[2] Wendel J.F. (2000). Genome evolution in polyploids. Plant. Mol. Biol.

[3] Bennett M.D. (2004). Perspectives on polyploidy in plants—Ancient and neo. Biol. J. Linn. Soc.

[4] Hendry A.P., Bolnick D.I., Berner D., Peichel C.L. (2009). Along the speciation continuum in sticklebacks. J. Fish. Biol.

[5] Soltis D.E., Buggs R.J.A., Doyle J.J., Soltis P.S. (2010). What we still don’t know about polyploidy. Taxon.

[6] Ramsey J., Schemske D.W. (1998). Pathways, mechanisms, and rates of polyploid formation in flowering plants. Annu. Rev. Ecol. Syst.

[7] Ramsey J., Schemske D.W. (2002). Neopolyploidy in flowering plants. Annu. Rev. Ecol. Syst.

[8] Carputo D., Frusciante L., Peloquin S.J. (2003). The role of 2*n* gametes and endosperm balance number in the origin and evolution of polyploids in the tuber-bearing Solanums. Genetics.

[9] Chen Z.J. (2007). Genetic and epigenetic mechanisms for gene expression and phenotypic variation in plant polyploids. Annu. Rev. Plant. Biol.

[10] Bretagnolle F., Thompson J.D. (1995). Tansley review no. 78. Gametes with the stomatic chromosome number: Mechanisms of their formation and role in the evolution of autopolypoid plants. New Phytol.

[11] D’Erfurth I., Jolivet S., Froger N., Catrice O., Novatchkova M., Simon M., Jenczewski E., Mercier R. (2008). Mutations in ATPS1 (*Arabidopsis Thaliana Parallel Spindle 1*) lead to the production of diploid pollen grains. PLoS Genet.

[12] Erilova A., Brownfield L., Exner V., Rosa M., Twell D., Scheid O.M., Hennig L., Köhler C. (2009). Imprinting of the polycomb group gene *MEDEA* serves as a ploidy sensor in *Arabidopsis*. PLoS Genet.

[13] Mercier R., Vezon D., Bullier E., Motamayor J.C., Sellier A., Lefèvre F., Pelletier G., Horlow C. (2001). SWITCH1 (SWI1): A novel protein required for the establishment of sister chromatid cohesion and for bivalent formation at meiosis. Genes Dev.

[14] Agashe B., Prasad C.K., Siddiqi I. (2002). Identification and analysis of *DYAD*: A gene required for meiotic chromosome organisation and female meiotic progression in *Arabidopsis*. Development.

[15] D’Erfurth I., Jolivet S., Froger N., Catrice O., Novatchkova M., Mercier R. (2009). Turning meiosis into mitosis. PLoS Biol.

[16] D’Erfurth I., Cromer L., Jolivet S., Girard C., Horlow C., Sun Y., To J.P.C., Berchowitz L.E., Copenhaver G.P., Mercier R. (2010). The CYCLIN-A CYCA1;2/TAM is required for the meiosis I to meiosis II transition and cooperates with OSD1 for the prophase to first meiotic division transition. PLoS Genet.

[17] Fawcett J.A., Maere S., van de Peer Y. (2009). Plants with double genomes might have had a better chance to survive the cretaceous-tertiary extinction event. Proc. Natl. Acad. Sci. USA.

[18] Comai L. (2005). The advantages and disadvantages of being polyploid. Nat. Rev. Genet.

[19] Osborn T.C., Pires J.C., Birchler J.A., Auger D.L., Chen Z.J., Lee H.-S., Comai L., Madlung A., Doerge R.W., Colot V. (2003). Understanding mechanisms of novel gene expression in polyploids. Trends Genet.

[20] Mayrose I., Zhan S.H., Rothfels C.J., Magnuson-Ford K., Barker M.S., Rieseberg L.H., Otto S.P. (2011). Recently formed polyploid plants diversify at lower rates. Science.

[21] Pires J.C., Lim K.Y., Kovarík A., Matyásek R., Boyd A., Leitch A.R., Leitch I.J., Bennett M.D., Soltis P.S., Soltis D.E. (2004). Molecular cytogenetic analysis of recently evolved *Tragopogon* (Asteraceae) allopolyploids reveal a karyotype that is additive of the diploid progenitors. Am. J. Bot.

[22] Szadkowski E., Eber F., Huteau V., Lode M., Huneau C., Belcram H., Coriton O., Manzanares-Dauleux M.J., Delourme R., King G.J. (2010). The first meiosis of resynthesized *Brassica napus*, a genome blender. New Phytol.

[23] Wang K., Guo W., Yang Z., Hu Y., Zhang W., Zhou B., Stelly D., Chen Z., Zhang T. (2010). Structure and size variations between 12A and 12D homoeologous chromosomes based on high-resolution cytogenetic map in allotetraploid cotton. Chromosoma.

[24] Chester M., Gallagher J.P., Symonds V.V., Cruz da Silva A.V., Mavrodiev E.V., Leitch A.R., Soltis P.S., Soltis D.E. (2012). Extensive chromosomal variation in a recently formed natural allopolyploid species, *Tragopogon miscellus* (Asteraceae). Proc. Natl. Acad. Sci. USA.

[25] Porceddu A., Albertini E., Barcaccia G., Falistocco E., Falcinelli M. (2002). Linkage mapping in apomictic and sexual Kentucky bluegrass (*Poa pratensis* L.) genotypes using a two way pseudo-testcross strategy based on AFLP and SAMPl markers. Theor. Appl. Genet.

[26] Gaeta R.T., Pires J.C., Iniguez-Luy F., Leon E., Osborn T.C. (2007). Genomic changes in resynthesized *Brassica napus* and their effect on gene expression and phenotype. Plant Cell.

[27] Nicolas S.D., Leflon M., Monod H., Eber F., Coriton O., Huteau V., Chèvre A.-M., Jenczewski E. (2009). Genetic regulation of meiotic cross-overs between related genomes in *Brassica napus* haploids and hybrids. Plant Cell.

[28] Cifuentes M., Eber F., Lucas M.-O., Lode M., Chèvre A.-M., Jenczewski E. (2010). Repeated polyploidy drove different levels of crossover suppression between homoeologous chromosomes in *Brassica napus* allohaploids. Plant Cell.

[29] Leitch I.J., Bennett M.D. (1997). Polyploidy in angiosperms. Trends Plant Sci.

[30] Liu B., Vega J.M., Feldman M. (1998). Rapid genomic changes in newly synthesized amphiploids of *Triticum* and *Aegilops*. II. Changes in low-copy coding DNA sequences. Genome.

[31] Madlung A., Tyagi A.P., Watson B., Jiang H., Kagochi T., Doerge R.W., Martienssen R., Comai L. (2005). Genomic changes in synthetic *Arabidopsis* polyploids. Plant J.

[32] Song K., Lu P., Tang K., Osborn T.C. (1995). Rapid genome change in synthetic polyploids of *Brassica* and its implications for polyploid evolution. Proc. Natl. Acad. Sci. USA.

[33] Salmon A., Ainouche M.L., Wendel J.F. (2005). Genetic and epigenetic consequences of recent hybridization and polyploidy in *Spartina* (Poaceae). Mol. Ecol.

[34] Wu K.K., Burnquist W., Sorrells M.E., Tew T.L., Moore P.H., Tanksley S.D. (1992). The detection and estimation of linkage in polyploids using single-dose restriction fragments. Theor. Appl. Genet.

[35] Hackett C.A., Bradshaw J.E., Meyer R.C., McNicol J.W., Milbourne D., Waugh R. (1998). Linkage analysis in tetraploid species: A simulation study. Genet. Res.

[36] Ripol M.I., Churchill G.A., da Silva J.A.G., Sorrells M. (1999). Statistical aspects of genetic mapping in autopolyploids. Gene.

[37] Wu R., Ma C.-X., Casella G. (2004). A bivalent polyploid model for mapping quantitative trait loci in outcrossing tetraploids. Genetics.

[38] Li J., Das K., Liu J., Fu G., Li Y., Tobias C., Wu R. (2012). Statistical models for genetic mapping in polyploids: Challenges and opportunities. Methods Mol. Biol.

[39] Ma J.F., Shen R., Zhao Z., Wissuwa M., Takeuchi Y., Ebitani T., Yano M. (2002). Response of rice to Al stress and identification of quantitative trait loci for Al tolerance. Plant Cell Physiol.

[40] Julier B., Flajoulot S., Barre P., Cardinet G., Santoni S., Huguet T., Huyghe C (2003). Construction of two genetic linkage maps in cultivated tetraploid alfalfa (*Medicago sativa*) using microsatellite and AFLP markers. BMC Plant Biol.

[41] Barcaccia G., Meneghetti S., Albertini E., Triest L., Lucchin M. (2003). Linkage mapping in tetraploid willows: Segregation of molecular markers and estimation of linkage phases support an allotetraploid structure for *Salix alba* × *Salix fragilis* interspecific hybrids. Heredity.

[42] Le Cunff L., Garsmeur O., Raboin L.M., Pauquet J., Telismart H., Selvi A., Grivet L., Philippe R., Begum D., Deu M. (2008). Diploid/polyploid syntenic shuttle mapping and haplotype-specific chromosome walking toward a rust resistance gene (*Bru1*) in highly polyploid sugarcane (2*n*~12x~115). Genetics.

[43] Esselink G.D., Nybom H., Vosman B. (2004). Assignment of allelic configuration in polyploids using the MAC-PR (microsatellite DNA allele counting—peak ratios) method. Theor. Appl. Genet.

[44] Van Dijk T., Noordijk Y., Dubos T., Bink M., Meulenbroek B., Visser R., van de Weg E (2012). Microsatellite allele dose and configuration establishment (MADCE): An integrated approach for genetic studies in allopolyploids. BMC Plant Biol.

[45] Oliver R., Jellen E., Ladizinsky G., Korol A., Kilian A., Beard J., Dumlupinar Z., Wisniewski-Morehead N., Svedin E., Coon M. (2011). New Diversity Arrays Technology (DArT) markers for tetraploid oat (*Avena magna* Murphy et Terrell) provide the first complete oat linkage map and markers linked to domestication genes from hexaploid *A. sativa* L. Theor. Appl. Genet.

[46] Reyna-López G.E., Simpson J., Ruiz-Herrera J. (1997). Differences in DNA methylation patterns are detectable during the dimorphic transition of fungi by amplification of restriction polymorphisms. Mol. Gen. Genet.

[47] Madlung A., Masuelli R.W., Watson B., Reynolds S.H., Davison J., Comai L. (2002). Remodeling of DNA methylation and phenotypic and transcriptional changes in synthetic Arabidopsis allotetraploids. Plant Physiol.

[48] Liu B., Brubaker C.L., Mergeai G., Cronn R.C., Wendel J.F. (2001). Polyploid formation in cotton is not accompanied by rapid genomic changes. Genome.

[49] Axelsson T., Bowman C.M., Sharpe A.G., Lydiate D.J., Lagercrantz U. (2000). Amphidiploid *Brassica juncea* contains conserved progenitor genomes. Genome.

[50] Jannoo N., Grivet L., Chantret N., Garsmeur O., Glaszmann J.C., Arruda P., D’Hont A. (2007). Orthologous comparison in a gene-rich region among grasses reveals stability in the sugarcane polyploid genome. Plant J.

[51] Paterson A.H. (2006). Leafing through the genomes of our major crop plants: Strategies for capturing unique information. Nat. Rev. Genet.

[52] Schranz M.E., Song B.-H., Windsor A.J., Mitchell-Olds T. (2007). Comparative genomics in the Brassicaceae: A family-wide perspective. Curr. Opin. Plant Biol.

[53] Margulies E.H., Birney E. (2008). Approaches to comparative sequence analysis: Towards a functional view of vertebrate genomes. Nat. Rev. Genet.

[54] Chantret N., Salse J., Sabot F., Rahman S., Bellec A., Laubin B., Dubois I., Dossat C., Sourdille P., Joudrier P. (2005). Molecular basis of evolutionary events that shaped the *hardness* locus in diploid and polyploid wheat species (Triticum and Aegilops). Plant Cell.

[55] Gao S., Gu Y., Wu J., Coleman-Derr D., Huo N., Crossman C., Jia J., Zuo Q., Ren Z., Anderson O. (2007). Rapid evolution and complex structural organization in genomic regions harboring multiple prolamin genes in the polyploid wheat genome. Plant Mol. Biol.

[56] Innes R.W., Ameline-Torregrosa C., Ashfield T., Cannon E., Cannon S.B., Chacko B., Chen N.W.G., Couloux A., Dalwani A., Denny R. (2008). Differential accumulation of retroelements and diversification of NB-LRR disease resistance genes in duplicated regions following polyploidy in the ancestor of soybean. Plant Physiol.

[57] Town C.D., Cheung F., Maiti R., Crabtree J., Haas B.J., Wortman J.R., Hine E.E., Althoff R., Arbogast T.S., Tallon L.J. (2006). Comparative genomics of *Brassica oleracea* and *Arabidopsis thaliana* reveal gene loss, fragmentation, and dispersal after polyploidy. Plant Cell.

[58] Li W., Huang L., Gill B.S. (2008). Recurrent deletions of puroindoline genes at the grain *hardness* locus in four independent lineages of polyploid wheat. Plant Physiol.

[59] De Bodt S., Maere S., van de Peer Y. (2005). Genome duplication and the origin of angiosperms. Trends Ecol. Evol.

[60] Soltis D.E., Bell C.D., Kim S., Soltis P.S. (2008). Origin and early evolution of angiosperms. Ann. N. Y. Acad. Sci.

[61] Velasco R., Zharkikh A., Affourtit J., Dhingra A., Cestaro A., Kalyanaraman A., Fontana P., Bhatnagar S.K., Troggio M., Pruss D. (2010). The genome of the domesticated apple (*Malus domestica* Borkh.). Nat. Genet.

[62] The Tomato Genome Consortium (2012). The tomato genome sequence provides insights into fleshy fruit evolution. Nature.

[63] Jiao Y., Wickett N.J., Ayyampalayam S., Chanderbali A.S., Landherr L., Ralph P.E., Tomsho L.P., Hu Y., Liang H., Soltis P.S. (2011). Ancestral polyploidy in seed plants and angiosperms. Nature.

[64] Levasseur A., Pontarotti P (2011). The role of duplications in the evolution of genomes highlights the need for evolutionary-based approaches in comparative genomics. Biol. Direct.

[65] Wawrzynski A., Ashfield T., Chen N.W.G., Mammadov J., Nguyen A., Podicheti R., Cannon S.B., Thareau V., Ameline-Torregrosa C., Cannon E. (2008). Replication of nonautonomous retroelements in soybean appears to be both recent and common. Plant Physiol.

[66] Kraitshtein Z., Yaakov B., Khasdan V., Kashkush K. (2010). Genetic and epigenetic dynamics of a retrotransposon after allopolyploidization of wheat. Genetics.

[67] Ainouche M., Fortune P., Salmon A., Parisod C., Grandbastien M.A., Fukunaga K., Ricou M., Misset M.T. (2009). Hybridization, polyploidy and invasion: Lessons from *Spartina* (Poaceae). Biol. Invasions.

[68] Beaulieu J., Jean M., Belzile F. (2009). The allotetraploid *Arabidopsis thaliana–Arabidopsis lyrata* subsp. *petraea* as an alternative model system for the study of polyploidy in plants. Mol. Genet. Genomics.

[69] Kaur S., Francki M.G., Forster J.W. (2012). Identification, characterization and interpretation of single-nucleotide sequence variation in allopolyploid crop species. Plant Biotechnol. J.

[70] Hamilton J.P., Buell R.C. (2012). Advances in plant genome sequencing. Plant J.

[71] Shulaev V., Sargent D.J., Crowhurst R.N., Mockler T.C., Folkerts O., Delcher A.L., Jaiswal P., Mockaitis K., Liston A., Mane S.P. (2011). The genome of woodland strawberry (*Fragaria vesca*). Nat. Genet.

[72] Bancroft I., Morgan C., Fraser F., Higgins J., Wells R., Clissold L., Baker D., Long Y., Meng J., Wang X. (2011). Dissecting the genome of the polyploid crop oilseed rape by transcriptome sequencing. Nat. Biotech.

[73] Paux E., Sourdille P., Salse J., Saintenac C., Choulet F., Leroy P., Korol A., Michalak M., Kianian S., Spielmeyer W. (2008). A physical map of the 1-gigabase bread wheat chromosome 3B. Science.

[74] Saintenac C., Jiang D., Akhunov E (2011). Targeted analysis of nucleotide and copy number variation by exon capture in allotetraploid wheat genome. Genome Biol.

[75] Akhunov E., Nicolet C., Dvorak J. (2009). Single nucleotide polymorphism genotyping in polyploid wheat with the Illumina GoldenGate assay. Theor. Appl. Genet.

[76] Allen A., Islamovic E., Kaur J., Gold S., Shah D., Smith T.J. (2011). Transgenic maize plants expressing the Totivirus antifungal protein, KP4, are highly resistant to corn smut. Plant Biotechnol. J.

[77] Lai J., Li R., Xu X., Jin W., Xu M., Zhao H., Xiang Z., Song W., Ying K., Zhang M. (2010). Genome-wide patterns of genetic variation among elite maize inbred lines. Nat. Genet.

[78] Bundock P.C., Eliott F.G., Ablett G., Benson A.D., Casu R.E., Aitken K.S., Henry R.J. (2009). Targeted single nucleotide polymorphism (SNP) discovery in a highly polyploid plant species using 454 sequencing. Plant Biotechnol. J.

[79] Buggs R.J., Chamala S., Wu W., Gao L., May G.D., Schnable P.S., Soltis D.E., Soltis P.S., Barbazuk W.B. (2010). Characterization of duplicate gene evolution in the recent natural allopolyploid *Tragopogon miscellus* by next-generation sequencing and Sequenom iPLEX MassARRAY genotyping. Mol. Ecol.

[80] Gabriel S., Ziaugra L., Tabbaa D (2009). SNP Genotyping Using the Sequenom iPLEX MassARRAY Platform. Current Protocols in Human Genetics.

[81] Han Y., Kang Y., Torres-Jerez I., Cheung F., Town C., Zhao P., Udvardi M., Monteros M (2011). Genome-wide SNP discovery in tetraploid alfalfa using 454 sequencing and high resolution melting analysis. BMC Genomics.

[82] Cho M.H., Ciulla D., Klanderman B.J., Raby B.A., Silverman E.K. (2008). High-resolution melting curve analysis of genomic and whole-genome amplified DNA. Clin. Chem.

[83] Han Y., Khu D.M., Monteros M.J. (2012). High-resolution melting analysis for SNP genotyping and mapping in tetraploid alfalfa (*Medicago sativa* L.). Mol. Breed.

[84] Salmon A., Flagel L., Ying B., Udall J.A., Wendel J.F. (2010). Homoeologous nonreciprocal recombination in polyploid cotton. New Phytol.

[85] Trick M., Long Y., Meng J., Bancroft I. (2009). Single nucleotide polymorphism (SNP) discovery in the polyploid *Brassica napus* using Solexa transcriptome sequencing. Plant Biotechnol. J.

[86] Guo M., Davis D., Birchler J.A. (1996). Dosage effects on gene expression in a maize ploidy series. Genetics.

[87] Comai L., Tyagi A.P., Winter K., Holmes-Davis R., Reynolds S.H., Stevens Y., Byers B. (2000). Phenotypic instability and rapid gene silencing in newly formed *Arabidopsis* allotetraploids. Plant Cell.

[88] Lee H.-S., Chen Z.J. (2001). Protein-coding genes are epigenetically regulated in *Arabidopsis* polyploids. Proc. Natl. Acad. Sci. USA.

[89] He P., Friebe B.R., Gill B.S., Zhou J.-M. (2003). Allopolyploidy alters gene expression in the highly stable hexaploid wheat. Plant Mol. Biol.

[90] Adams K.L., Cronn R., Percifield R., Wendel J.F. (2003). Genes duplicated by polyploidy show unequal contributions to the transcriptome and organ-specific reciprocal silencing. Proc. Natl. Acad. Sci. USA.

[91] Tate J.A., Ni Z., Scheen A.-C., Koh J., Gilbert C.A., Lefkowitz D., Chen Z.J., Soltis P.S., Soltis D.E. (2006). Evolution and expression of homeologous loci in *Tragopogon miscellus* (Asteraceae), a recent and reciprocally formed allopolyploid. Genetics.

[92] Bottley A., Xia G.M., Koebner R.M.D. (2006). Homoeologous gene silencing in hexaploid wheat. Plant J.

[93] Schena M., Shalon D., Davis R.W., Brown P.O. (1995). Quantitative monitoring of gene expression patterns with a complementary DNA microarray. Science.

[94] Chagué V., Just J., Mestiri I., Balzergue S., Tanguy A.-M., Huneau C., Huteau V., Belcram H., Coriton O., Jahier J. (2010). Genome-wide gene expression changes in genetically stable synthetic and natural wheat allohexaploids. New Phytol.

[95] Pumphrey M., Bai J., Laudencia-Chingcuanco D., Anderson O., Gill B.S. (2009). Nonadditive expression of homoeologous genes is established upon polyploidization in hexaploid wheat. Genetics.

[96] Chaudhary B., Hovav R., Flagel L., Mittler R., Wendel J (2009). Parallel expression evolution of oxidative stress-related genes in fiber from wild and domesticated diploid and polyploid cotton (*Gossypium*). BMC Genomics.

[97] Marmagne A., Brabant P., Thiellement H., Alix K. (2010). Analysis of gene expression in resynthesized B*rassica napus* allotetraploids: Transcriptional changes do not explain differential protein regulation. New Phytol.

[98] Chelaifa H., Monnier A., Ainouche M. (2010). Transcriptomic changes following recent natural hybridization and allopolyploidy in the salt marsh species *Spartina* × *townsendii* and *Spartina anglica* (Poaceae). New Phytol.

[99] Buggs R.J.A., Doust A.N., Tate J.A., Koh J., Soltis K., Feltus F.A., Paterson A.H., Soltis P.S., Soltis D.E. (2009). Gene loss and silencing in *Tragopogon miscellus* (Asteraceae): Comparison of natural and synthetic allotetraploids. Heredity.

[100] Doyle J.J., Flagel L.E., Paterson A.H., Rapp R.A., Soltis D.E., Soltis P.S., Wendel J.F. (2008). Evolutionary genetics of genome merger and doubling in plants. Annu. Rev. Genet.

[101] Yu Z., Haberer G., Matthes M., Rattei T., Mayer K.F.X., Gierl A., Torres-Ruiz R.A. (2010). Impact of natural genetic variation on the transcriptome of autotetraploid *Arabidopsis thaliana*. Proc. Natl. Acad. Sci. USA.

[102] Stupar R.M., Bhaskar P.B., Yandell B.S., Rensink W.A., Hart A.L., Ouyang S., Veilleux R.E., Busse J.S., Erhardt R.J., Buell C.R. (2007). Phenotypic and transcriptomic changes associated with potato autopolyploidization. Genetics.

[103] Riddle N., Jiang H., An L., Doerge R., Birchler J. (2010). Gene expression analysis at the intersection of ploidy and hybridity in maize. Theor. Appl. Genet.

[104] Havecker E.R. (2011). Detection of small RNAs and microRNAs using deep sequencing technology. Methods Mol. Biol.

[105] Ha M., Lu J., Tian L., Ramachandran V., Kasschau K.D., Chapman E.J., Carrington J.C., Chen X., Wang X.-J., Chen Z.J. (2009). Small RNAs serve as a genetic buffer against genomic shock in *Arabidopsis* interspecific hybrids and allopolyploids. Proc. Natl. Acad. Sci. USA.

[106] Ng D.W.-K., Zhang C., Miller M., Palmer G., Whiteley M., Tholl D., Chen Z.J. (2011). *Cis*- and *trans*-regulation of miR163 and target genes confers natural variation of secondary metabolites in two *Arabidopsis* species and their allopolyploids. Plant Cell.

[107] Wang Z., Gerstein M., Snyder M. (2009). RNA-Seq: A revolutionary tool for transcriptomics. Nat. Rev. Genet.

[108] Croate E.C., Doyle J.J. (2010). Quantifying whole transcriptome size, a prerequisite for understanding transcriptome evolution across species: An example from a plant allopolyploid. Genome Biol. Evol.

[109] Ilut D.C., Coate J.E., Luciano A.K., Owens T.G., May G.D., Farmer A., Doyle J.J. (2012). A comparative transcriptomic study of an allotetraploid and its diploid progenitors illustrates the unique advantages and challenges of RNA-seq in plant species. Am. J. Bot.

[110] Birchler J.A., Newton K.J. (1981). Modulation of protein levels in chromosomal dosage series of maize: The biochemical basis of aneuploid syndromes. Genetics.

[111] Islam N., Tsujimoto H., Hirano H. (2003). Proteome analysis of diploid, tetraploid and hexaploid wheat: Towards understanding genome interaction in protein expression. Proteomics.

[112] Albertin W., Balliau T., Brabant P., Chèvre A.-M., Eber F., Malosse C., Thiellement H. (2006). Numerous and rapid nonstochastic modifications of gene products in newly synthesized *Brassica napus* allotetraploids. Genetics.

[113] Albertin W., Langella O., Joets J., Négroni L., Zivy M., Damerval C., Thiellement H. (2009). Comparative proteomics of leaf, stem, and root tissues of synthetic *Brassica napus*. Proteomics.

[114] Song X., Ni Z., Yao Y., Xie C., Li Z., Wu H., Zhang Y., Sun Q. (2007). Wheat (*Triticum aestivum* L.) root proteome and differentially expressed root proteins between hybrid and parents. Proteomics.

[115] Yao H., Kato A., Mooney B., Birchler J. (2011). Phenotypic and gene expression analyses of a ploidy series of maize inbred Oh43. Plant Mol. Biol.

[116] Ng D.W.K., Zhang C., Miller M., Shen Z., Briggs S.P., Chen Z.J. (2012). Proteomic divergence in Arabidopsis autopolyploids and allopolyploids and their progenitors. Heredity.

[117] Elshire R.J., Glaubitz J.C., Sun Q., Poland J.A., Kawamoto K., Buckler E.S., Mitchell S.E. (2011). A robust, simple genotyping-by-sequencing (GBS) approach for high diversity species. PLoS One.

[118] Grover C.E., Salmon A., Wendel J.F. (2012). Targeted sequence capture as a powerful tool for evolutionary analysis. Am. J. Bot.

[119] Cronn R., Knaus B.J., Liston A., Maughan P.J., Parks M., Syring J.V., Udall J. (2012). Targeted enrichment strategies for next-generation plant biology. Am. J. Bot.

[120] Kvam V.M., Liu P., Si Y. (2012). A comparison of statistical methods for detecting differentially expressed genes from RNA-seq data. Am. J. Bot.

[121] Ercolano M.R. (2012).

[122] Schmutz J., Cannon S.B., Schlueter J., Ma J., Mitros T., Nelson W., Hyten D.L., Song Q., Thelen J.J., Cheng J. (2010). Genome sequence of the palaeopolyploid soybean. Nature.

[123] Potato Genome Sequencing Consortium (2012). Genome sequence and analysis of the tuber crop potato. Nature.

